# Effect of saffron on liver metastases in patients suffering from cancers with liver metastases: A randomized, double blind, placebo-controlled clinical trial

**Published:** 2015

**Authors:** Azar Hosseini, Seyed Hamed Mousavi, Anis Ghanbari, Fatemeh Homaee Shandiz, Hamid Reza Raziee, Masoud Pezeshki Rad, Seyed Hadi Mousavi

**Affiliations:** 1 *Pharmacological Research Center of Medicinal Plants, School of Medicine, Mashhad University of Medical Sciences, Mashhad, Iran*; 2 *Department of Pharmacology, School of Medicine, Mashhad University of Medical Sciences, Mashhad, Iran*; 3 *Department of Oncology, Omid Hospitsal, Mashhad, Iran*; 4 *Department of Oncology, Emam Reza Hospitsal, Mashhad, Iran*; 5 *Medical Toxicology Research Center, School of Medicine, Mashhad University of Medical Sciences, Mashhad, Iran*

**Keywords:** *Saffron*, *Cancer*, *Liver Metastases*

## Abstract

**Objective:**

Cancer represents the second cause of mortality in the world. Saffron as a medicinal plant is known for its anti-cancer and anti-depressant properties. In this randomized double blind clinical trial, the effects of saffron on response to treatment in patients suffering from liver metastasis were evaluated.

**Materials and Methods:**

Thirteen patients suffering from liver metastases who referred to Ghaem and Imam Reza hospital, Mashhad, Iran were included in this study and then divided into two different groups. Both groups received chemotherapy regimen. Patients in group one were treated with saffron capsule (50 mg, twice daily) during chemotherapy periods whereas patients in group two received placebo. A sum of the longest diameter were calculated and compared for all lesions in IV contrast CT scan before and after the treatment.

**Results:**

from 13 patients included in this study, six patients quit and seven continued until the end. In saffron-treated group, two patients showed partial and complete response (50%) whereas in placebo group, no response was seen. Also, two deaths in placebo and one in saffron group occurred.

**Conclusion:**

This research suggests that saffron might be useful in patients suffering from liver metastasis. However, further investigations with larger sample size are required.

## Introduction

Nowadays, cancer is a major health problem which is considered as the second cause of death after myocardial infarction (Dalkic et al., 2010 ▶). Different methods for treatment of cancer are including chemotherapy, surgery and radiotherapy or combination of them. Chemotherapy is one of the principal modes of the treatment of cancer patients (Liu 2009 ▶). Although the goal of chemotherapy is elimination of tumor cells, it influences normal cells and leads to many adverse eﬀects in multiple organ systems (Zhou et al., 2007 ▶). 

However, the goal of researchers is to apply nutritional or supplementing agents to reduce side effects or enhance the efficacy of chemotherapeutic drugs (Davis et al., 2007 ▶). 

Different studies have shown that some antioxidant compounds increase tumor response to chemotherapy, reduce adverse effects of anti-cancer drugs or relieve the toxicity of chemotherapeutics on normal cells (Lamson and Brignall 1999 ▶). Recent studies have shown that various plant-derived agents like genistein, epigallocatechin gallate (EGCG), curcumin, resveratrol, indole-3-carbinol, and pro-anthocyanidin improved the eﬃcacy of traditional chemotherapeutic agents (Prasad et al., 2001 ▶; Davis et al., 2007 ▶) . However, herbal medicine could be effective in cancer treatment. 

Saffron is produced from dried stigma of *Crocus sativus* L. (Iridaceae) which is cultivated in Azerbaijan, France, Greece, India and Iran (Abdullaev and Espinosa-Aguirre 2004 ▶). Saffron contains volatile and non-volatile compounds which are effective in the treatment of different diseases (Basker andNegbi, 1983 ▶). Studies have shown that saffron and its constituents are effective in cancer therapy (Abdullaev and Espinosa-Aguirre 2004 ▶).

Topical administration of saffron extract inhibited initiation and promotion of DMBA, a skin-specific carcinogen (Das et al., 2010 ▶). Also, saffron extract reduced the toxic effects of cisplatin in mice and prolonged the life span (Nair, 1991 ▶). Saffron extract reduced some of toxic effects of anticancer drugs on blood factors (Nair, 1991 ▶). It decreased the nephrotoxicity of cisplatin, alone or in combination with other antioxidants (Daly, 1998 ▶).

Also, *in vitro* studies have shown that saffron and its constituents are effective against various cancer cells including colorectal (HCT-116, SW-480, and HT-29) (Aung et al., 2007 ▶) and breast cancer cells (MCF-7 and MDA-MB-231) (Chryssanthi et al., 2007 ▶), non-small cell lung cancer (NSCLC) cells (Aung et al., 2007 ▶), lung adenocarcinoma cells (A549), lung fibroblasts cells (WI-38), VA-13 cells (WI-38 cells transformed in vitro by SV40 tumor virus) (Abdullaev and Frenkel, 1992 ▶; Abdullaev and Espinosa-Aguirre 2004 ▶) , lung cancer-bearing mice (Magesh et al., 2006 ▶), skin carcinogenesis in mice (Salomi et al., 1991 ▶; Konoshima et al., 1998 ▶), leukemia cells (HL-60), osteosarcoma, fibrosarcoma [4, 12], ovarian carcinoma (Abdullaev and Espinosa-Aguirre 2004 ▶; Aung et al., 2007 ▶), and cervical epithelioid carcinoma cells (HeLa) (Abdullaev and Espinosa-Aguirre 2004 ▶; Surh et al., 2005 ▶). Saffron significantly inhibited the growth of colorectal cancer cells without affecting normal cells (Aung et al., 2007 ▶). Also, it had inhibitory effects on breast cancer cells in a dose-dependent manner (Chryssanthi et al., 2007 ▶). Crocetin decreased the growth of three malignant human cell lines including HeLa, A549, and VA13 (Surh et al., 2005 ▶). Overall, *in vivo* and *in vitro* studies have shown that saffron inhibits tumor growth, alone or in combination with other treatments (Hosseinzadeh et al., 2007 ▶; Schmidt et al., 2007 ▶). According to beneficial effects of saffron against cancer cells shown by *in vivo* and *in vitro* studies, we assumed that it may be effective in a clinical setting. In this study we investigated the anti-cancer effect of saffron in combination with chemotherapy in cancer patients suffering from liver metastatic, for the first time.

## Materials and Methods

Here we conducted a randomized, double blind, placebo-controlled clinical trial from May 2009 to October 2010. Participants were recruited from patients referred to oncology clinic of Ghaem and Imam Reza hospitals, Mashhad, Iran for at least 10 months. These patients had liver metastasis with primary cancer including esophagus, stomach, colon, ovarian or breast. At first, an oncologist diagnosed type of cancer and administrated chemotherapeutic agents. If patients tend to participate in this study, they were asked to write an informed consent. In the next step, the patient was introduced to another colleague to receive either saffron or placebo (containing starch) capsules. This study was approved by the Ethics Committee of Mashhad University of Medical Sciences with Grant number: 87432. Pregnant patients were excluded from the study.

**Plant material**

*Crocus sativus* L. stigma was obtained from Novin Saffron Co. (Mashhad, Iran). It was formulated as capsules containing 50 mg dried saffron stigma.

**Sample size**

Thirteen subjects with liver metastasis participated in this clinical trial. But, only seven patients (one male and six females) continued the study and other patients quit (three subjects died and three subjects had problems to continue). The patients had breast cancer (n=4), esophagus cancer (n=1), ovarian cancer (n=1) and colon cancer (n=1). The patients were divided into two groups:

Saffron group: Subjects (n=4) received chemotherapy for 6 courses (each course was continued for 3 weeks) and took 100 mg saffron (50 mg, twice daily in the morning and afternoon).

Placebo group: Subjects (n=3) received their chemotherapy treatment similar to first group but they took 100 mg starch as placebo (50 mg, twice daily in the morning and afternoon).

Patient 1: A 45-year-old woman with breast cancer in saffron group; Patient 2: A 76-year-old man with esophagus cancer in saffron group; Patient 3: A 45-year-old woman with breast cancer in placebo group; Patient 4: A 37-year-old woman with breast cancer in saffron group; Patient 5: A 40-year-old woman with breast cancer in saffron group; Patient 6: A 38-year-old woman with colon cancer in placebo group and Patient 7: A 56-year-old woman with ovarian cancer in placebo group (Table 1). Abdominal CT scans (before and after the study) were compared for each patient. 

**Table1:** Descriptive characteristics of the participants

**Patient **	**Gender**	**Cancer type**	**Age**	**Group**
**1**	Woman	Breast cancer	45	Saffron
**2**	Man	esophagus cancer	76	Saffron
**3**	Woman	Breast cancer	45	Placebo
**4**	Woman	Breast cancer	37	Saffron
**5**	Woman	Breast cancer	40	Saffron
**6**	Woman	Colon cancer	38	Placebo
**7**	Woman	Ovarian cancer	56	Placebo

**Evaluation of Response to Treatment**

The number and size of metastatic lesions were calculated according to guideline of National Cancer Institute; In this method, the longest diameter of tumor was measured. According to this method decreased, increased or unchanged metastatic lesions was defined in 4 groups (Bamshad et al., 2006 ▶):

Complete response: All metastatic lesions are eliminated. 

Partial response: 30% reduction in the total size of metastatic lesions.

Progressive disease: 20% increase in the total size of metastatic lesions. 

Stable disease: a state between progressive and partial response.

**Statistical Analysis**

The number and size of tumor were compared by Paired t-test. Also, the data were compared with control group by unpaired t-test.

## Results

**Figure1:**
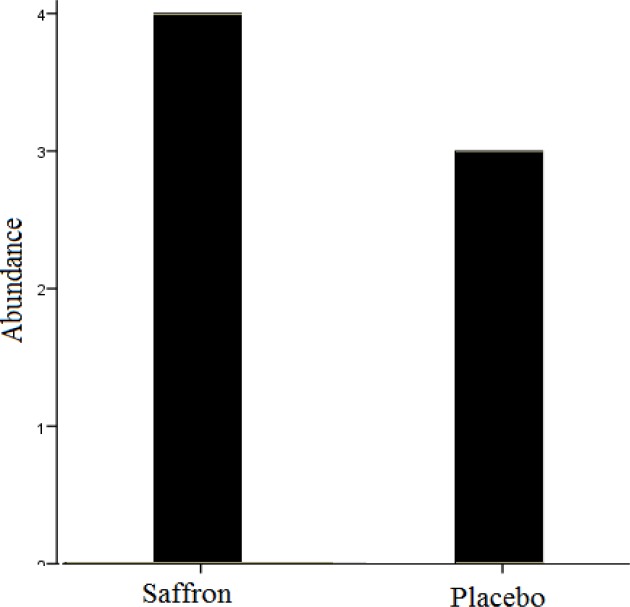
The abundance of patients in placebo and saffron groups

The participants’ mean age was 47 years old. Placebo and saffron groups included 3 and 4 patients, respectively. The abundance of each group was shown as histogram (Figure1).

The patients were evaluated according to CT scan results. 

Treatment with saffron led to complete (n=1), partial (n=1), progressive (n=1) and stable (n=1) responses. But, in placebo group, complete and partial responses were not seen and results included unchanged (n=2) and progressive (n=1) responses (Table 2).

**Table 2:** Type of response to treatment in saffron and placebo groups

	**Complete**	**Partial**	**Stable**	**Progressive**
**Saffron**	1	1	1	1
**Placebo**	-	-	2	1

In total, results revealed 28.6% progressive (n=2), 42.9% stable (n=3), 14.3% partial (n=1) and 14.3% complete responses.

**Figure 2:**
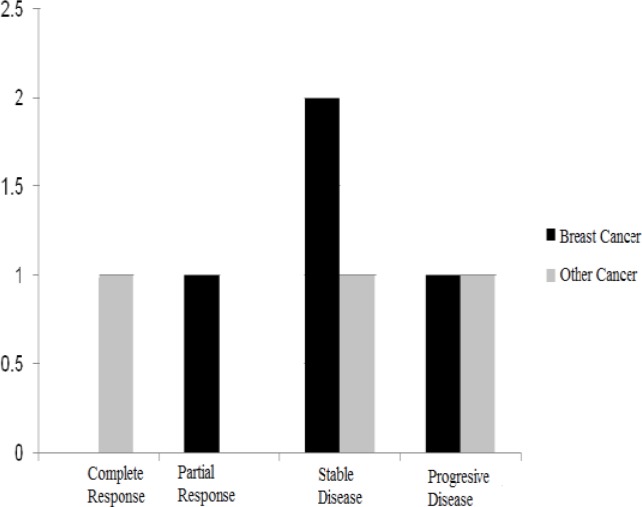
Type of response to treatment in breast and other breast cancers

Figure 2 compares type of response to treatment between breast and other cancer patients.

Partial response (n=1), stable (n=2) and progressive disease (n=1) were observed in breast cancer patients, whereas in other cancers, complete response (n=1, esophagus cancer), progressive disease (n=1, colon cancer) and stable disease (n=1, ovarian cancer) were observed. Complete response was observed in the male subject whereas partial, stable and progressive responses were observed in female patients. The serious side effect such as anaphylaxis shock was not observed in saffron and placebo groups.

## Discussion

Cancer is a complex disease and affects different tissues and organs. According to global statistics, every year about 7 million people die due to cancer (Dalkic et al., 2010 ▶). The treatment methods include surgery, radiotherapy and chemotherapy. In initial stage, chemotherapy with anti-cancer drugs is the selective method (Liu 2009 ▶). Unfortunately, most of anti-cancer drugs cause side effects such as fatigue, nausea, vomiting, diarrhea, mucositis, pain, rashes, infections, headaches, etc. Also, most of them affect normal cells and lead to disturbance in other organs (Loprinzi et al., 2007 ▶). Previous studies have shown that phytotherapy could be effective in cancer treatment. Plants contain different compounds from which, some can improve the effectiveness of the chemotherapeutic drugs or reduce their side effects. Hence, addition of these compounds to daily regimen of cancer patients may be helpful (Davis et al., 2007 ▶). Some of plant-derived agents like curcumin, resveratrol, withania somnifera, grean tea and comptothecin have shown beneficial effects on different types of cancer in clinical trials (Dhillon et al., 2008 ▶; Nguyen et al., 2009 ▶; Biswal et al., 2013 ▶; Choan et al., 2005 ▶; Hsu, 1980 ▶). Anti-cancer properties of saffron have been shown, *in vivo* and *in vitro*. In this study, saffron anti-cancer effects were investigated in a randomized, double blind, placebo-controlled clinical trial. Our findings showed that 50% of subjects in saffron group responded to treatment as complete or partial responses which were not observed in placebo group. This result may be related to saffron anti-cancer effect that has been revealed when administered alone or in combination with anti-cancer drugs (Daly, 1998 ▶). In placebo group, two patients died whereas in saffron group, one patient died. Previous studies have shown that saffron prolonged life span of mice (Nair, 1991 ▶). However, less mortality rate in saffron group in comparison with placebo may be related to prolonging life span by saffron. *In vivo* and *in vitro* studies have shown anti-cancer effects of saffron. Some of constituents of saffron are carotenoids. Crocin as a water-soluble carotenoid is responsible for saffron color. Picrocrocin, a degradation product of the zeaxanthin carotenoid is responsible for the bitter taste of saffron and also is a precursor of safranal (D’Alessandro et al., 2013 ▶; Tavakkol-Afshari et al., 2008 ▶). Animal studies and cultured human malignant cell lines have demonstrated anti-tumor and anti-cancer activities of saffron and its constituents (Carmona et al., 2007 ▶; Nair et al., 1991 ▶; Mousavi et al., 2009 ▶). Different studies have shown that crocin inhibit tumor growth in rats with colorectal cancer (García-Olmo et al., 1999 ▶). Crocin may be a safer alternative to treat ATRA-sensitive cancer in women of childbearing age with promyelocytic leukemia and other ATRA-responsive cancers (Martin et al., 2002 ▶). Microscopic studies have shown that crocin induce apoptosis in HeLa cell line (Mousavi et al., 2011 ▶), also protects the bladder against cyclophosphamide-induced toxicity (Jnaneshwari et al., 2013 ▶). Anti-cancer mechanisms of saffron and its constituents include inhibition of DNA and RNA synthesis (Chryssanthi et al., 2011 ▶), inhibition of ROS production (Premkumar et al., 2003 ▶), involvement in the metabolic conversion of carotenoids to retinoids (Garc´ıa-L´opez et al., 2012 ▶), promotion of interactions mediated via lectins and elevation of sulfhydryl levels in cells (Moln´ar et al., 2009 ▶). Previous studies have shown that carotenoids have anti-cancer properties (Tanaka and Shnimizu, 2012 ▶; Firdous et al., 2010 ▶) and anti-cancer effects of saffron may be due to its carotenoid constituents. According to our results, saffron may be useful in cancer treatment but further investigations with larger sample size are required.

## Conflict of interest

The authors declare that they have no conflict of interest.
